# Three Decades of Internet- and Computer-Based Interventions for the Treatment of Depression: Protocol for a Systematic Review and Meta-Analysis

**DOI:** 10.2196/14860

**Published:** 2020-03-24

**Authors:** Isaac Moshe, Yannik Terhorst, Pim Cuijpers, Ioana Cristea, Laura Pulkki-Råback, Lasse Sander

**Affiliations:** 1 Department of Psychology and Logopedics Faculty of Medicine University of Helsinki Helsinki Finland; 2 Department of Research Methods University of Ulm Ulm Germany; 3 Department of Clinical, Neuro-, and Developmental Psychology Amsterdam Public Health Research Institute Vrije Universiteit Amsterdam Amsterdam Netherlands; 4 Department of Clinical Psychology and Psychotherapy Babes-Bolyai University Cluj-Napoca Romania; 5 Research Centre for Child Psychiatry University of Turku Turku Finland; 6 Department of Rehabilitation Psychology and Psychotherapy University of Freiburg Freiburg Germany

**Keywords:** depression, internet-based interventions, meta-analysis, review

## Abstract

**Background:**

Depression is one of the leading causes of disability worldwide. Internet- and computer-based interventions (IBIs) have been shown to provide effective, scalable forms of treatment. More than 100 controlled trials and a growing number of meta-analyses published over the past 30 years have demonstrated the efficacy of IBIs in reducing symptoms in the short and long term. Despite the large body of research, no comprehensive review or meta-analysis has been conducted to date that evaluates how the effectiveness of IBIs has evolved over time.

**Objective:**

This systematic review and meta-analysis aims to evaluate whether there has been a change in the effectiveness of IBIs on the treatment of depression over the past 30 years and to identify potential variables moderating the effect size.

**Methods:**

A sensitive search strategy will be executed across the Cochrane Central Register of Controlled Trials (CENTRAL), MEDLINE, EMBASE, and PsycINFO. Data extraction and evaluation will be conducted by two independent researchers. Risk of bias will be assessed. A multilevel meta-regression model will be used to analyze the data and estimate effect size.

**Results:**

The search was completed in mid-2019. We expect the results to be submitted for publication in early 2020.

**Conclusions:**

The year 2020 will mark 30 years since the first paper was published on the use of IBIs for the treatment of depression. Despite the large and rapidly growing body of research in the field, evaluations of effectiveness to date are missing the temporal dimension. This review will address that gap and provide valuable analysis of how the effectiveness of interventions has evolved over the past three decades; which participant-, intervention-, and study-related variables moderate changes in effectiveness; and where research in the field may benefit from increased focus.

**Trial Registration:**

PROSPERO CRD42019136554; https://www.crd.york.ac.uk/prospero/display_record.php?RecordID=136554

**International Registered Report Identifier (IRRID):**

PRR1-10.2196/14860

## Introduction

### Background

Depression is one of the leading causes of disability worldwide, with global prevalence rates estimated at 4.7% [1,2]. Depression has been identified as a risk factor for many chronic health conditions [3], is associated with poor quality of life [4], has a significant burden of fatal and nonfatal disease [5], and a highly negative economic impact [6].

Cognitive behavioral therapy (CBT) is the most widely practiced and researched form of psychotherapy for depression, with an extensive body of research supporting its efficacy in reducing depressive symptoms [7,8].

Despite the demonstrated efficacy, the World Health Organization estimates that approximately 34 million people suffering from major depressive disorder go untreated each year in Europe and the United States alone, representing a treatment gap of more than 56% [9]. Barriers to effective care include difficulty accessing a nearby provider, the prohibitive cost of treatment, lack of insurance and trained health care providers, long waiting list times, and the social stigma associated with mental disorders [10,11].

The development of internet- and computer-based therapy has provided an effective method of meeting some of these challenges. Emerging in 1990, the first version of computer-based CBT (cCBT) was effectively a CBT manual delivered via a CD-ROM [12]. However, with the development and widespread adoption of the internet in the 1990s, internet delivery became the norm [13].

During an internet- or computer-based intervention (IBI), patients typically log in to a website to read, watch, hear, and download materials arranged into a series of lessons or modules. They receive homework assignments and regularly complete computer-administered questionnaires relevant to their presenting problems, allowing a therapist or other support person to monitor their progress and outcomes [14,15].

There are a number of advantages offered by IBIs over traditional forms of face-to-face therapy [14]. First, in the case of online interventions, the ability for patients to access IBIs at anytime and from anywhere with an internet connection significantly lowers the barrier to access. Second, the anonymity of IBIs allows patients to circumvent the stigma surrounding mental disorders, which prevents many from even mentioning their problems when consulting general practitioners. Finally, the time savings associated with internet-delivered therapy has enabled health care providers to increase the delivery of therapy and reduce wait-list times, making it a highly scalable form of therapy.

Over the past 30 years, IBIs have been developed and tested for a range of mental disorders, the most common of which are anxiety and depression disorders [16]. Interventions have employed a variety of therapeutic approaches—from CBT [16] to acceptance and commitment therapy [17], psychodynamic approaches [18], and interpersonal psychotherapy [19].

### Effectiveness of Internet- and Computer-Based Interventions

The most widely researched type of IBI is internet-delivered cognitive behavior therapy (iCBT), with more than 100 randomized controlled trials (RCTs) and a growing number of effectiveness studies [20-22] and meta-analyses [8,23-35] demonstrating its efficacy. One of the earliest meta-analyses by Spek et al [23] found a moderate posttreatment effect size across 12 RCTs for participants with depression compared with control groups. Subsequent reviews have reported similar findings, with pooled standardized mean differences ranging from Cohen *d*=.32 [23] to Hedges’ *g*=0.78 [25] for interventions compared with placebo, treatment as usual, and wait-list. A particularly important comparison for IBIs is traditional, face-to-face therapy. Although there have been few trials to date, a meta-analysis by Carlbring et al [35] indicated that there was no significant difference between iCBT and face-to-face treatments, a finding supported by Webb et al [36]. In addition to RCTs, a number of effectiveness studies have demonstrated that iCBT can be effectively delivered in routine clinical practice, with effects similar to those observed in efficacy trials [21,22]. One notable (although widely debated) exception to these findings was the large-scale REEACT (Randomised Evaluation of the Effectiveness and Acceptability of Computerised Therapy) trial, which compared two iCBT interventions (Beating the Blues and MoodGYM) with usual general practitioner care in a primary care setting in the in which it was concluded that the benefits of IBIs demonstrated in efficacy trials may not transfer to clinical settings [37].

Finally, small but significant effect size superiority has been shown at both 3- to 6- and 9- to 18-month follow-ups indicating the potential for IBIs to deliver sustained benefits over time [26].

Despite these positive findings, the growing number of RCTs and meta-analyses conducted to date only provide a pooled estimate of effect size at a singular point in time. To the best of our knowledge, no research has been published that evaluates how the effectiveness of IBIs has evolved over time. That is to say, have outcomes improved, deteriorated, or remained the same? Therefore, the primary aim of the proposed study is to examine the effect of time on the effectiveness of IBIs in the treatment of depression, in which the term “effectiveness” will be used to encompass both efficacy and effectiveness trials [38]. As a result of significant advances in digital technology over the past 30 years, together with a greater understanding of the moderators of change in IBIs, we hypothesize that the effectiveness of IBIs has increased with time.

### Factors Influencing the Effectiveness of Internet- and Computer-Based Interventions

As the field of IBIs has developed over the past 30 years, an increasing number of studies have looked at the factors influencing effectiveness. Researchers have identified a broad range of factors related to (1) participant characteristics, (2) intervention components, and (3) study design and quality.

Regarding participant characteristics, marital status, education level, gender, and pretreatment depression severity have all been shown to influence outcomes [39-42]. However, it is important to note that methodological and power limitations may affect the reliability of these findings, which may also account for the considerable variability found across studies [43,44]. In a small but growing number of trials looking at age, IBIs have also been shown to be effective in the treatment of depression for both children and older adults [45,46].

When it comes to intervention components, IBIs vary considerably in the therapeutic approach adopted, the design of the platform, the content used, and the mode of delivery. Perhaps the most significant and consistent finding regarding the impact of intervention components on effectiveness is the role of human support or “guidance,” in which a number of RCTs and meta-analyses have demonstrated that guided interventions lead to greater effect sizes than unguided interventions [15,32,33,44,47]. Additional research has also studied the impact of the amount of guidance received (the dose-response relationship), the qualification of those providing guidance (eg, therapist versus administrative personnel), the communication mode (email, phone, video chat) [15,32,34], and the acceptability of guided and unguided interventions compared with other delivery formats [47].

Another important factor influencing the reported effectiveness of IBIs is study design and quality. Researchers have long been aware of the substantial heterogeneity between studies in the field, leading to inconsistent effect sizes. In recent years, concerns over a number of methodological shortcomings affecting much of the research have been raised. A meta-analysis by Richards and Richardson [15], for example, revealed a high risk of missing data in RCTs as well as possible publication bias. In an in-depth review of study design and quality, Arnberg et al [48] reported on the lack of proper quality assessment and objective outcome measures, the paucity of noninferiority trials, the relatively small sample sizes in most trials, a focus on short-term outcomes, the failure to report on deterioration and adverse events, and the overrepresentation of trials conducted in a limited number of countries threatening generalizability. The type of control used in the study has also been shown to have a significant impact on effectiveness. As Webb et al [36] pointed out, the majority of iCBT studies have used a wait-list as their control condition, which a number of researchers have demonstrated leads to a significantly greater effect size than care as usual [49-52]. If this is indeed the case, research would benefit considerably from understanding whether any potential increase in the effectiveness of IBIs over the past decades is a result of an improvement in the interventions themselves or simply a result of changes in study design and quality. In so doing, we would also reveal how methodological standards in the field have developed over time, exposing potential shortcomings that need to be addressed.

### Aims and Objectives

Using a meta-regression analysis, this study will examine whether there has been a change in the effectiveness of IBIs for the treatment of depression over the past 30 years independent of study-related moderating variables. It will also describe relevant developments in the field over time (eg, populations studied, changes in intervention design, study quality, and sample size).

## Methods

This protocol has been developed in line with the PRISMA (Preferred Reporting Items for Systematic Review and Meta-Analysis) protocols statement [53]. The systematic review and meta-analysis has been registered with the PROSPERO (International Prospective Register of Systematic Reviews) database (registration number: CRD42019136554).

### Eligibility Criteria

In accordance with the PRISMA checklist recommendations, this review will use the participants, interventions, comparators, and outcome(s) process for framing and reporting the review criteria, and the study design of the included studies will be reported (Textbox 1).

**PICOS (participants, interventions, comparators, outcomes, and study design) elements of the study inclusion criteria.**

**Participants**

- Individuals of all age groups and gender with depressive symptoms

**Interventions**

- Internet- and computer-based psychological interventions (IBI) (eg, IBI with guidance, IBI without guidance)

**Comparators**

- Wait-list
- Treatment as usual
- Attention control
- No treatment

**Outcomes**

- Symptom-specific: depression severity
- Intervention-related: acceptability

**Study design**

- Randomized controlled trials published in peer-reviewed journals

#### Participants

We will include studies of people of all age groups and genders with depressive symptoms. No restrictions regarding ethnicity and cultural background will be applied.

#### Interventions

Included studies must report on one or more interventions that are based on psychological interventions. A multitude of different psychological interventions are available. Following Kampling et al [54], we differentiate between (1) CBT (eg, problem solving), (2) psychodynamic psychotherapy (eg, psychoanalytic therapy), (3) behavior therapy or behavior modification (eg, exposure therapy), (4) systemic therapy (eg, family therapy), (5) third-wave CBTs (eg, acceptance and commitment therapy), (6) humanistic therapies (eg, Rogerian therapy), (7) integrative therapies (eg, interpersonal therapy) and other psychological-oriented interventions (eg, bibliotherapy) [54].

Interventions must be provided via a computer or mobile device (eg, tablet or mobile phone) in either an offline or online setting, defined as computerized-, online-, internet-, or Web-based. Interventions that are delivered solely via mobile apps will be excluded due to differences in the way they approach diagnosis and delivery of the intervention compared with IBIs, as well as significant heterogeneity between the apps themselves (eg, the use of ecological momentary assessments, duration of tasks or modules, and use of conversational agents) [14,55]. We will include both guided and unguided interventions. Guided interventions will refer to interventions that are primarily based on self-help material but accompanied by some form of minimal human guidance related to the therapeutic content. In line with Karyotaki et al [44], guidance will be considered minimal if it is provided at low intervals and through electronic means, such as email, phone, and online messaging (eg, brief email feedback on weekly homework). We will consider an intervention unguided if it is self-help with no human guidance or support relating to the therapeutic content. Studies involving “blended therapy” (where computerized therapy is combined with face-to-face therapy) will be excluded because the therapeutic support here differs substantially from minimal therapeutic contact provided in guided interventions.

#### Comparators

We will include all RCTs with an inactive control condition (eg, treatment as usual, attention control condition, wait-list control, or no treatment). Studies will be excluded if they compared the intervention to an active control (eg, face-to-face therapy or pharmacotherapy). In cases of multiple comparators or multiple interventions and one comparator in one study, all comparisons between intervention group(s) and comparator(s) will be included separately. We will use a three-level meta-regression model to account for dependencies (see Data Analysis).

#### Outcomes

The primary outcome will be effect size in depressive symptomatology measured by validated self- or clinician-rated depression scales. Multiple effect sizes (eg, multiple outcomes or multiple groups) will be included separately. Resulting dependencies will be accounted for in the three-level meta-analysis [56].

Intention-to-treat data, if available, will be used for the primary analysis. Secondary analyses will be reported for per-protocol data. The per-protocol analysis will be based on the sample of participants who adequately adhered to the intervention protocol by completing at least 80% of sessions [57].

As a secondary outcome, we will include acceptability of treatment, operationalized as the proportion of patients who left the study early for any reason during the acute phase of treatment [58].

#### Study Design

Parallel RCTs will be included. Published, peer-reviewed, full-text articles in all languages will be included.

### Predictors and Moderators

This study will investigate the following potential moderators of effect size over time: (1) pretreatment depression severity, (2) guidance, (3) comorbidities, (4) control group, and (5) study quality. Time will be operationalized as the year of publication.

Pretreatment depression severity will be operationalized using the sum score of a validated rating scale. Preference will be given to the measure reported by the majority of the included studies. In studies using different outcome measures, the score will be converted into the most commonly used scale using the established conversion algorithms [59]. If this approach does not cover a substantial proportion of the obtained data, scale scores will be transformed into *z* scores to create a standardized common metric for pretreatment depression severity [34,60].

Guidance will be operationalized as being either “guided” or “unguided” interventions. *Guided* interventions will be defined as support related to the therapeutic content provided by a human at low intervals during the intervention and delivered through electronic means, such as email, phone, and online chat. *Unguided* will be defined as an intervention that that does not provide support related to the therapeutic content but may involve support related to the intervention itself (eg, instruction on how to use the program) and/or automated feedback. “Blended” interventions or those involving any face-to-face support during the intervention will be excluded from either definition.

Comorbidities will be defined as target populations with a comorbid somatic disorder. Control group will be operationalized according to the Comparators section and study quality according to the Risk of Bias section.

### Study Identification and Selection

Relevant articles will be identified according to the following steps. First, a database search will be conducted using a sensitive search strategy for the Cochrane Central Register of Controlled Trials (CENTRAL), PsycINFO, EMBASE, and MEDLINE. The sensitivity of the strategy will be validated a priori using a sample set of articles from previous meta-analyses. In a second step, studies included in reference lists of relevant existing systematic reviews and meta-analyses will be checked for eligibility. In a third step, a hand search will be conducted of the reference lists of all included studies.

In the case of missing data, we will contact trialists for information on unpublished or ongoing studies, or to request additional trial data and determine eligibility for inclusion in this review. The search will be restricted to studies published in the 30 years from January 1990 to April 2019.

**Figure 1:**
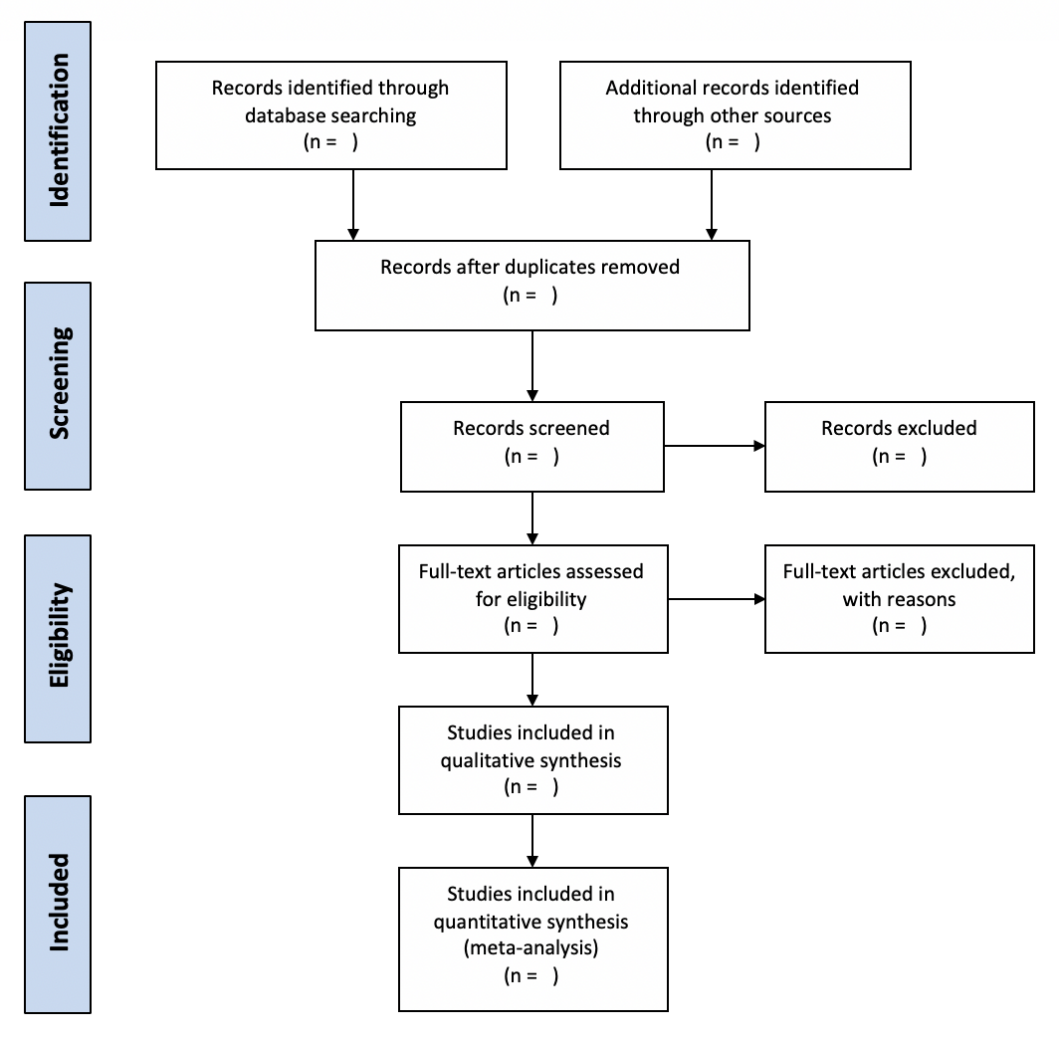
PRISMA flow diagram.

The selection of articles will be conducted by two independent reviewers (IM and IC). In the first step, they will screen all titles and abstracts yielded by the database search. In the second step, full texts of the selected articles will be retrieved and screened in terms of the aforementioned eligibility criteria. Disagreement will be resolved by a discussion among the reviewers. When needed to resolve a disagreement, a third reviewer (LS) will be consulted. We will identify and exclude duplicate records, and we will collate multiple reports that relate to the same study so that each study rather than each report is the unit of interest in the review. We will record all decisions made during the selection process in sufficient detail with numbers of studies and references to complete a PRISMA flow diagram and “characteristics of included studies” and “characteristics of excluded studies” tables at the end of the review (see Figure 1).

### Data Collection

We will use a data extraction spreadsheet to extract study characteristics and outcome data. Two review authors (IM and IC) will extract study characteristics and outcome data from the included studies. Individuals involved in the data extraction process will not be blinded regarding study authors, journal, or institution. We will extract the following study characteristics:

1. Study identification items: description of trial, including primary researcher and year of publication;
2. Study design: sample size, methodology, target mental disorders, control group, and duration of intervention;
3. Study setting: nationality, environment (community, primary care, secondary care), and specific population groups (eg, worker population, students, diabetes patients);
4. Participants: N, mean age, gender, primary diagnosis, comorbid diagnoses, and severity of condition;
5. Interventions: therapeutic theoretical approach (eg, CBT), guidance (guided or unguided); and
6. Outcome measures: primary and secondary outcomes as listed in Main Outcomes and Additional Outcomes herein.

### Risk of Bias (Quality) Assessment

To evaluate the quality of the research, two independent reviewers will assess the risk of bias for each study using the criteria outlined in the risk of bias tool for randomized trials [61,62]. Any disagreement will be resolved by a discussion among the reviewers. When needed to resolve a disagreement, a third researcher (LS) will be consulted.

Risk of bias will be assessed in the following domains: (1) selection bias, (2) performance bias, (3) detection bias, (4) attrition bias, (5) reporting bias, and (6) other bias. Risk of bias in each domain will be judged as “low,” “unclear,” or “high.” We will summarize the risk of bias judgments across different studies for each of the domains listed. Overall risk of bias will be derived based on the risk of bias tool for randomized trials; a score of low=1, unclear=2, and high=3 will be given for each domain. The sum of all domain scores will be used as the overall risk of bias indicator.

Study heterogeneity will be calculated with the *I*^2^ statistic. A random-effects model will be assumed. A value of 0% indicates no heterogeneity; higher values indicate higher heterogeneity. A heterogeneity of 25% is defined as the threshold for low, 50% for moderate, and 75% for high [63]. To account for uncertainty, 95% confidence intervals will be calculated for *I*^2^. In addition, predictive intervals will be reported to estimate the range of the true effect [64].

We will use a funnel plot and Q-Q plot to detect potential biasing effects. Asymmetry will be tested using the Egger test [65].

### Strategy for Data Analysis and Synthesis

#### Interrater Reliability

Cohen kappa will be calculated to assess interrater reliability for categorical variables and intraclass correlation for continuous variables [66,67]. Disagreements will be solved by discussion.

#### Effect Sizes

Cohen *d* will be used for between-group effect size [68]: Differences in groups’ means will be divided by their pooled standard deviation. If *d* is not reported, available coefficients (eg, *r*) will be transformed or *d* will be calculated based on given information (eg, *t* value, N, *F* value, beta). If insufficient information is provided for the calculation, the corresponding author will be contacted [69].

#### Quantitative Data Synthesis and Statistical Calculations

The meta-analytic effect size will be estimated using a three-level meta-regression model with random effects [70,71]. By assuming a three-level structure, we account for three different variance components distributed over the three levels in the model. This includes sampling variance of the extracted effect sizes at level one, variance between the extracted effect sizes from the same study at level two, and variance between studies at level three [56]. The three-level model will be compared with a two-level model by information criteria to evaluate the need for a three-level structure.

To investigate the development in effect size over time, the primary moderator of interest will be time. Both linear and quadratic time trends will be assumed. In addition, moderators, as outlined previously, will be inserted in the model. Two regression models will be used: (1) a parsimonious model, in which only single meta-regression significant predictors are included (“parsimonious model”) and (2) a model including all predictors. Models will be compared using information criteria.

All analyses will be conducted using R [72]. The package “metafor” will be used as the primary analysis package [71,73].

## Results

The search was completed after submitting the protocol in mid-2019. Data analysis was completed at the end of 2019. We expect the results to be submitted for publication in early 2020.

## Discussion

This systematic review will address a significant lack of research examining the field of IBIs for the treatment of depression from a temporal perspective. Specifically, it will provide valuable analysis of how the effectiveness of interventions has changed over time and identify relevant moderators and study characteristics that may be related to possible changes in efficacy. This is especially important given the rapid rise in internet-based interventions for the treatment of depression over the past decade and the challenges that exist in treating the enormous disease burden of depression.

To the best of our knowledge, this is the first planned study that will review the field of IBIs from a temporal perspective. In so doing, it will shed light on where research has been both over- and underfocused over the past three decades, alert researchers and funding bodies to important research questions that have not been given sufficient attention, and expose methodological shortcomings affecting research to date, thus providing valuable guidance on next steps for the field.

## Abbreviations

CBT: cognitive behavioral therapy
IBI: internet- and computer-based intervention
iCBT: internet-based cognitive behavioral therapy
PRISMA: Preferred Reporting Items for Systematic Reviews and Meta-Analyses
PROSPERO: International Prospective Register of Systematic Reviews
RCT: randomized controlled trial
REEACT: Randomised Evaluation of the Effectiveness and Acceptability of Computerised Therapy

## References

[1] Whiteford HA, Degenhardt L, Rehm J, Baxter AJ, Ferrari AJ, Erskine HE, Charlson FJ, Norman RE, Flaxman AD, Johns N, Burstein R, Murray CJL, Vos T (2013). Global burden of disease attributable to mental and substance use disorders: findings from the Global Burden of Disease Study 2010. Lancet.

[2] Ferrari AJ, Charlson FJ, Norman RE, Patten SB, Freedman G, Murray CJL, Vos T, Whiteford HA (2013). Burden of depressive disorders by country, sex, age, and year: findings from the global burden of disease study 2010. PLoS Med.

[3] Krishnan KRR, Delong M, Kraemer H, Carney R, Spiegel D, Gordon C, McDonald W, Dew M, Alexopoulos G, Buckwalter K, Cohen PD, Evans D, Kaufmann PG, Olin J, Otey E, Wainscott C (2002). Comorbidity of depression with other medical diseases in the elderly. Biol Psychiatry.

[4] Saarni SI, Suvisaari J, Sintonen H, Pirkola S, Koskinen S, Aromaa A, Lönnqvist J (2007). Impact of psychiatric disorders on health-related quality of life: general population survey. Br J Psychiatry.

[5] Jia H, Zack MM, Thompson WW, Crosby AE, Gottesman II (2015). Impact of depression on quality-adjusted life expectancy (QALE) directly as well as indirectly through suicide. Soc Psychiatry Psychiatr Epidemiol.

[6] Greenberg PE, Birnbaum HG (2005). The economic burden of depression in the US: societal and patient perspectives. Expert Opin Pharmacother.

[7] Hofmann SG, Asnaani A, Vonk IJJ, Sawyer AT, Fang A (2012). The efficacy of cognitive behavioral therapy: a review of meta-analyses. Cognit Ther Res.

[8] Cuijpers P, Berking M, Andersson G, Quigley L, Kleiboer A, Dobson KS (2013). A meta-analysis of cognitive-behavioural therapy for adult depression, alone and in comparison with other treatments. Can J Psychiatry.

[9] Kohn R, Saxena S, Levav I, Saraceno B (2004). The treatment gap in mental health care. Bull World Health Organ.

[10] Saxena S, Thornicroft G, Knapp M, Whiteford H (2007). Resources for mental health: scarcity, inequity, and inefficiency. Lancet.

[11] Andrade LH, Alonso J, Mneimneh Z, Wells JE, Al-Hamzawi A, Borges G, Bromet E, Bruffaerts R, de Girolamo G, de Graaf R, Florescu S, Gureje O, Hinkov HR, Hu C, Huang Y, Hwang I, Jin R, Karam EG, Kovess-Masfety V, Levinson D, Matschinger H, O'Neill S, Posada-Villa J, Sagar R, Sampson NA, Sasu C, Stein DJ, Takeshima T, Viana MC, Xavier M, Kessler RC (2014). Barriers to mental health treatment: results from the WHO World Mental Health surveys. Psychol Med.

[12] Selmi PM, Klein MH, Greist JH, Sorrell SP, Erdman HP (1990). Computer-administered cognitive-behavioral therapy for depression. Am J Psychiatry.

[13] Andersson G, Titov N (2014). Advantages and limitations of Internet-based interventions for common mental disorders. World Psychiatry.

[14] Ebert DD, Van Daele T, Nordgreen T, Karekla M, Compare A, Zarbo C, Brugnera A, Øverland S, Trebbi G, Jensen KL, Kaehlke F, Baumeister H (2018). Internet- and mobile-based psychological interventions: applications, efficacy, and potential for improving mental health: a report of the EFPA E-Health Taskforce. Eur Psychol.

[15] Richards D, Richardson T (2012). Computer-based psychological treatments for depression: a systematic review and meta-analysis. Clin Psychol Rev.

[16] Andersson G (2009). Using the Internet to provide cognitive behaviour therapy. Behav Res Ther.

[17] Brown M, Glendenning A, Hoon AE, John A (2016). Effectiveness of web-delivered acceptance and commitment therapy in relation to mental health and well-being: a systematic review and meta-analysis. J Med Internet Res.

[18] Johansson R, Frederick RJ, Andersson G (2013). Using the internet to provide psychodynamic psychotherapy. Psychodyn Psychiatry.

[19] Donker T, Bennett K, Bennett A, Mackinnon A, van Straten A, Cuijpers P, Christensen H, Griffiths KM (2013). Internet-delivered interpersonal psychotherapy versus internet-delivered cognitive behavioral therapy for adults with depressive symptoms: randomized controlled noninferiority trial. J Med Internet Res.

[20] Andersson G (2016). Internet-delivered psychological treatments. Annu Rev Clin Psychol.

[21] Titov N, Dear BF, Staples LG, Bennett-Levy J, Klein B, Rapee RM, Shann C, Richards D, Andersson G, Ritterband L, Purtell C, Bezuidenhout G, Johnston L, Nielssen OB (2015). MindSpot Clinic: an accessible, efficient, and effective online treatment service for anxiety and depression. Psychiatr Serv.

[22] Hedman E, Ljótsson B, Kaldo V, Hesser H, El Alaoui S, Kraepelien M, Andersson E, Rück C, Svanborg C, Andersson G, Lindefors N (2014). Effectiveness of Internet-based cognitive behaviour therapy for depression in routine psychiatric care. J Affect Disord.

[23] Spek V, Cuijpers P, Nyklícek I, Riper H, Keyzer J, Pop V (2007). Internet-based cognitive behaviour therapy for symptoms of depression and anxiety: a meta-analysis. Psychol Med.

[24] Andersson G, Cuijpers P (2009). Internet-based and other computerized psychological treatments for adult depression: a meta-analysis. Cogn Behav Ther.

[25] Andrews G, Cuijpers P, Craske MG, McEvoy P, Titov N (2010). Computer therapy for the anxiety and depressive disorders is effective, acceptable and practical health care: a meta-analysis. PLoS One.

[26] Andrews G, Basu A, Cuijpers P, Craske MG, McEvoy P, English CL, Newby JM (2018). Computer therapy for the anxiety and depression disorders is effective, acceptable and practical health care: An updated meta-analysis. J Anxiety Disord.

[27] Barak A, Hen L, Boniel-Nissim M, Shapira N (2008). A comprehensive review and a meta-analysis of the effectiveness of internet-based psychotherapeutic interventions. J Technol Hum Serv.

[28] Cuijpers P, van Straten A, Andersson G, van Oppen P (2008). Psychotherapy for depression in adults: a meta-analysis of comparative outcome studies. J Consult Clin Psychol.

[29] Cuijpers P, Andersson G, Donker T, van Straten A (2011). Psychological treatment of depression: results of a series of meta-analyses. Nord J Psychiatry.

[30] Cuijpers P, Geraedts AS, van Oppen P, Andersson G, Markowitz JC, van Straten A (2011). Interpersonal psychotherapy for depression: a meta-analysis. Am J Psychiatry.

[31] Cuijpers P, Donker T, Johansson R, Mohr DC, van Straten A, Andersson G (2011). Self-guided psychological treatment for depressive symptoms: a meta-analysis. PLoS One.

[32] Baumeister H, Reichler L, Munzinger M, Lin J (2014). The impact of guidance on Internet-based mental health interventions—a systematic review. Internet Interv.

[33] Königbauer J, Letsch J, Doebler P, Ebert D, Baumeister H (2017). Internet- and mobile-based depression interventions for people with diagnosed depression: a systematic review and meta-analysis. J Affect Disord.

[34] Karyotaki E, Riper H, Twisk J, Hoogendoorn A, Kleiboer A, Mira A, Mackinnon A, Meyer B, Botella C, Littlewood E, Andersson G, Christensen H, Klein JP, Schröder J, Bretón-López J, Scheider J, Griffiths K, Farrer L, Huibers MJ, Phillips R, Gilbody S, Moritz S, Berger T, Pop V, Spek V, Cuijpers P (2017). Efficacy of self-guided internet-based cognitive behavioral therapy in the treatment of depressive symptoms: a meta-analysis of individual participant data. JAMA Psychiatry.

[35] Carlbring P, Andersson G, Cuijpers P, Riper H, Hedman-Lagerlöf E (2018). Internet-based vs. face-to-face cognitive behavior therapy for psychiatric and somatic disorders: an updated systematic review and meta-analysis. Cogn Behav Ther.

[36] Webb CA, Rosso IM, Rauch SL (2017). Internet-based cognitive-behavioral therapy for depression: current progress and future directions. Harv Rev Psychiatry.

[37] Gilbody S, Littlewood E, Hewitt C, Brierley G, Tharmanathan P, Araya R, Barkham M, Bower P, Cooper C, Gask L, Kessler D, Lester H, Lovell K, Parry G, Richards DA, Andersen P, Brabyn S, Knowles S, Shepherd C, Tallon D, White D, REEACT Team (2015). Computerised cognitive behaviour therapy (cCBT) as treatment for depression in primary care (REEACT trial): large scale pragmatic randomised controlled trial. BMJ.

[38] Singal AG, Higgins PDR, Waljee AK (2014). A primer on effectiveness and efficacy trials. Clin Transl Gastroenterol.

[39] Button KS, Wiles NJ, Lewis G, Peters TJ, Kessler D (2012). Factors associated with differential response to online cognitive behavioural therapy. Soc Psychiatry Psychiatr Epidemiol.

[40] Warmerdam L, Van Straten A, Twisk J, Cuijpers P (2013). Predicting outcome of Internet-based treatment for depressive symptoms. Psychother Res.

[41] Donker T, Batterham PJ, Warmerdam L, Bennett K, Bennett A, Cuijpers P, Griffiths KM, Christensen H (2013). Predictors and moderators of response to internet-delivered Interpersonal Psychotherapy and Cognitive Behavior Therapy for depression. J Affect Disord.

[42] Spek V, Nyklícek I, Cuijpers P, Pop V (2008). Predictors of outcome of group and internet-based cognitive behavior therapy. J Affect Disord.

[43] Andersson G (2018). Internet interventions: past, present and future. Internet Interv.

[44] Karyotaki E, Ebert DD, Donkin L, Riper H, Twisk J, Burger S, Rozental A, Lange A, Williams AD, Zarski AC, Geraedts A, van Straten A, Kleiboer A, Meyer B, Ünlü Ince BB, Buntrock C, Lehr D, Snoek FJ, Andrews G, Andersson G, Choi I, Ruwaard J, Klein JP, Newby JM, Schröder J, Laferton JAC, Van Bastelaar K, Imamura K, Vernmark K, Boß L, Sheeber LB, Kivi M, Berking M, Titov N, Carlbring P, Johansson R, Kenter R, Perini S, Moritz S, Nobis S, Berger T, Kaldo V, Forsell Y, Lindefors N, Kraepelien M, Björkelund C, Kawakami N, Cuijpers P (2018). Do guided internet-based interventions result in clinically relevant changes for patients with depression? An individual participant data meta-analysis. Clin Psychol Rev.

[45] Spek V, Nyklícek I, Smits N, Cuijpers P, Riper H, Keyzer J, Pop V (2007). Internet-based cognitive behavioural therapy for subthreshold depression in people over 50 years old: a randomized controlled clinical trial. Psychol Med.

[46] Ebert DD, Zarski A, Christensen H, Stikkelbroek Y, Cuijpers P, Berking M, Riper H (2015). Internet and computer-based cognitive behavioral therapy for anxiety and depression in youth: a meta-analysis of randomized controlled outcome trials. PLoS One.

[47] Cuijpers P, Noma H, Karyotaki E, Cipriani A, Furukawa TA (2019). Effectiveness and acceptability of cognitive behavior therapy delivery formats in adults with depression: a network meta-analysis. JAMA Psychiatry.

[48] Arnberg FK, Linton SJ, Hultcrantz M, Heintz E, Jonsson U (2014). Internet-delivered psychological treatments for mood and anxiety disorders: a systematic review of their efficacy, safety, and cost-effectiveness. PLoS One.

[49] Cuijpers P, van Straten A, Bohlmeijer E, Hollon SD, Andersson G (2010). The effects of psychotherapy for adult depression are overestimated: a meta-analysis of study quality and effect size. Psychol Med.

[50] Watts SE, Turnell A, Kladnitski N, Newby JM, Andrews G (2015). Treatment-as-usual (TAU) is anything but usual: a meta-analysis of CBT versus TAU for anxiety and depression. J Affect Disord.

[51] Hempel S, Suttorp MJ, Miles JN, Wang Z, Maglione M, Morton S, Johnsen B, Valentine D, Shekelle PG (2011). Empirical Evidence of Associations Between Trial Quality and Effect Size.

[52] Zhang Z, Xu X, Ni H (2013). Small studies may overestimate the effect sizes in critical care meta-analyses: a meta-epidemiological study. Crit Care.

[53] Moher D, Liberati A, Tetzlaff J, Altman DG, PRISMA Group (2009). Preferred reporting items for systematic reviews and meta-analyses: the PRISMA statement. J Clin Epidemiol.

[54] Kampling H, Baumeister H, Jackel W, Mittag O (2014). Prevention of depression in chronically physically ill adults. Cochrane Database Syst Rev.

[55] Chan S, Godwin H, Gonzalez A, Yellowlees PM, Hilty DM (2017). Review of use and integration of mobile apps into psychiatric treatments. Curr Psychiatry Rep.

[56] Assink M, Wibbelink C (2016). Fitting three-level meta-analytic models in R: a step-by-step tutorial. Quant Method Psychol.

[57] van Ballegooijen W, Cuijpers P, van Straten A, Karyotaki E, Andersson G, Smit JH, Riper H (2014). Adherence to Internet-based and face-to-face cognitive behavioural therapy for depression: a meta-analysis. PLoS One.

[58] Cipriani A, Furukawa TA, Salanti G, Chaimani A, Atkinson LZ, Ogawa Y, Leucht S, Ruhe HG, Turner EH, Higgins JPT, Egger M, Takeshima N, Hayasaka Y, Imai H, Shinohara K, Tajika A, Ioannidis JPA, Geddes JR (2018). Comparative efficacy and acceptability of 21 antidepressant drugs for the acute treatment of adults with major depressive disorder: a systematic review and network meta-analysis. Lancet.

[59] Wahl I, Löwe B, Bjorner JB, Fischer F, Langs G, Voderholzer U, Aita SA, Bergemann N, Brähler E, Rose M (2014). Standardization of depression measurement: a common metric was developed for 11 self-report depression measures. J Clin Epidemiol.

[60] Furukawa TA, Karyotaki E, Suganuma A, Pompoli A, Ostinelli EG, Cipriani A, Cuijpers P, Efthimiou O (2019). Dismantling, personalising and optimising internet cognitive-behavioural therapy for depression: a study protocol for individual participant data component network meta-analysis. BMJ Open.

[61] Higgins JP, Altman DG, Gøtzsche PC, Jüni P, Moher D, Oxman AD, Savovic J, Schulz KF, Weeks L, Sterne JA, Cochrane BM, Cochrane SM (2011). The Cochrane Collaboration's tool for assessing risk of bias in randomised trials. BMJ.

[62] Higgins JPT, Thompson SG, Deeks JJ, Altman DG (2003). Measuring inconsistency in meta-analyses. BMJ.

[63] Ioannidis JPA, Patsopoulos NA, Evangelou E (2007). Uncertainty in heterogeneity estimates in meta-analyses. BMJ.

[64] IntHout J, Ioannidis JP, Rovers MM, Goeman JJ (2016). Plea for routinely presenting prediction intervals in meta-analysis. BMJ Open.

[65] Egger M, Davey Smith G, Schneider M, Minder C (1997). Bias in meta-analysis detected by a simple, graphical test. BMJ.

[66] Viera AJ, Garrett JM (2005). Understanding interobserver agreement: the kappa statistic. Fam Med.

[67] Fleiss JL (1986). The Design and Analysis of Clinical Experiments.

[68] Cohen J (1988). Statistical Power Analysis for the Behavioral Sciences.

[69] Polanin JR, Snilstveit B (2016). Converting between effect sizes. Campbell Syst Rev.

[70] Cooper H, Hedges LV, Cooper H, Hedges L, Valentine J (2009). Research synthesis as a scientific process. The Handbook of Research Synthesis and Meta-Analysis.

[71] Pastor DA, Lazowski RA (2018). On the multilevel nature of meta-analysis: a tutorial, comparison of software programs, and discussion of analytic choices. Multivariate Behav Res.

[72] R Development Core Team (2016). R: A language and environment for statistical computing. R Found Stat Comput.

[73] Viechtbauer W (2010). Conducting meta-analyses in R with the metafor package. J Stat Soft.

